# Research on Orchard Navigation Line Recognition Method Based on U-Net

**DOI:** 10.3390/s25226828

**Published:** 2025-11-07

**Authors:** Ning Xu, Xiangsen Ning, Aijuan Li, Zhihe Li, Yumin Song, Wenxuan Wu

**Affiliations:** 1 College of Agricultural Engineering and Food Science, Shandong University of Technology, Zibo 255000, China; xuning@saas.ac.cn; 2 Shandong Academy of Agricultural Machinery Sciences, Jinan 252100, China; wuwenxuan@saas.ac.cn; 3 Shandong Key Laboratory of Intelligent Agricultural Equipment in Hilly and Mountainous Areas, Jinan 250100, China; 4 School of Automotive Engineering, Shandong Jiaotong University, Jinan 250357, China; 13933028705@163.com (X.N.); liaijuan@sdjtu.edu.cn (A.L.); songyumin@sdjtu.edu.cn (Y.S.)

**Keywords:** orchard environment, navigation line, U-Net network, attention mechanism, divisible driving area

## Abstract

Aiming at the problems of complex image background and numerous interference factors faced by visual navigation systems in orchard environments, this paper proposes an orchard navigation line recognition method based on U-Net. Firstly, the drivable areas in the collected images are labeled using Labelme (a graphical tool for image annotation) to create an orchard dataset. Then, the Spatial Attention (SA) mechanism is inserted into the downsampling stage of the traditional U-Net semantic segmentation method, and the Coordinate Attention (CA) mechanism is added to the skip connection stage to obtain complete context information and optimize the feature restoration process of the drivable area in the field, thereby improving the overall segmentation accuracy of the model. Subsequently, the improved U-Net network is trained using the enhanced dataset to obtain the drivable area segmentation model. Based on the detected drivable area segmentation mask, the navigation line information is extracted, and the geometric center points are calculated row by row. After performing sliding window processing and bidirectional interpolation filling on the center points, the navigation line is generated through spline interpolation. Finally, the proposed method is compared and verified with U-Net, SegViT, SE-Net, and DeepLabv3+ networks. The results show that the improved drivable area segmentation model has a Recall of 90.23%, a Precision of 91.71%, a mean pixel accuracy (mPA) of 87.75%, and a mean intersection over union (mIoU) of 84.84%. Moreover, when comparing the recognized navigation line with the actual center line, the average distance error of the extracted navigation line is 56 mm, which can provide an effective reference for visual autonomous navigation in orchard environments.

## 1. Introduction

Navigation line recognition, as one of the key technologies for autonomous driving of agricultural tractors, is widely applied in tasks such as picking, spraying, and weeding [1,2,3], which can enhance agricultural productivity and address the issue of labor shortage. Common navigation techniques include LiDAR navigation, GNSS (Global Navigation Satellite System), and machine vision navigation [4]. The orchard environment is typically characterized by dense planting conditions. The canopy can block satellite positioning signals, leading to inaccurate positioning information [5]. LiDAR acquires point cloud information of the surrounding environment through beam scanning, but it has problems such as excessive redundant information, difficulty in feature extraction, and high equipment costs. Compared with LiDAR-based GNSS positioning technology, visual navigation has the advantages of rich semantic information, wide detection range, and controllable cost, making it one of the current research hotspots both at home and abroad. Moreover, navigation line recognition serves as a fundamental step for path tracking control, directly influencing the accuracy and stability of autonomous navigation [6]. Reliable line detection provides essential geometric and positional information for subsequent trajectory tracking and motion planning, which are critical for safe and efficient autonomous operation. Therefore, improvements in line recognition can contribute to enhancing overall control performance [7].

At present, the machine vision navigation in orchards mostly relies on the structure of the orchard and the characteristics of the trees to calculate the navigation lines by fitting the tree row lines with the tree root points. Ma Chi et al. [8] proposed a navigation feature target detection method based on deep learning, where the bottom center point of the target box replaces the tree root points. The tree row lines are extracted using cubic spline interpolation, and the navigation lines are generated by fitting using the least squares method. Shubo et al. [9] takes the midpoint of the tree trunk root as the navigation positioning base point, uses the improved YOLOv7 model to obtain the positioning reference points for the tree row lines on both sides, and then fits the tree row lines and the navigation lines using the least squares method. Xiao Ke et al. [10] proposed a navigation line calculation method based on Mask R-CNN and a tree row line calculation method based on the random sample consensus method.

The above research obtained navigation line information by directly extracting the boundary of the drivable area or by fitting tree row lines. It performed well in specific environments, but was susceptible to interference from factors such as varying tree shapes, complex environmental backgrounds, and occlusion of tree trunk and root points. Due to the low height and dense tree canopy of the fruit trees, it is difficult to identify the tree trunks. The road edges are irregular, making it difficult to stably generate road information. The method of fitting the tree row lines on both sides using the root points of the fruit trees is not suitable for complex orchard paths.

In view of the limitations of the existing methods, this paper proposes an orchard path navigation recognition method based on U-Net. By optimizing the network structure, the adaptability to environmental changes is enhanced. The downsampling part inserts the Spatial Attention mechanism, and the Coordinate Attention mechanism is added in the skip connection to obtain complete context information. It effectively deals with the complex orchard environment, and quickly and accurately extracts the drivable area of the orchard. The geometric center points are calculated row by row, and the center points are processed by sliding window and bidirectional interpolation filling. After that, spline interpolation is used to calculate and generate the navigation line, providing effective perception data for visual autonomous navigation in orchard environments.

The main contributions of this paper are summarized as follows:

(1) A complete orchard dataset was constructed using Labelme to label drivable areas, and data augmentation techniques such as brightness adjustment and Gaussian blur were applied to enhance the robustness and generalization ability of the model.
(2) An improved U-Net-based method for orchard navigation line recognition was designed by integrating the Spatial Attention mechanism in the downsampling stage and the Coordinate Attention mechanism in the skip connections, enabling the model to effectively extract key spatial features and fuse contextual information under complex orchard conditions.
(3) A navigation line extraction process based on drivable area segmentation was developed, in which geometric center points are calculated row by row, followed by sliding window smoothing, bidirectional interpolation, and spline fitting to generate a continuous and smooth navigation path.
(4) A multi-dimensional validation plan combining simulation and comparative experiments was implemented to evaluate the performance of the proposed method. The improved model achieved higher segmentation accuracy and navigation line precision than U-Net, SegViT, SE-Net, and DeepLabv3+, with an average navigation line error of only 56 mm, confirming its feasibility and practical value in orchard visual navigation.

The research content of this paper is as follows: Section 1 introduces the research background, the limitations of existing methods, and the research content of this study. Section 2 describes the creation of the dataset and the improved U-Net network structure. Section 3 presents the training and validation process of the model, as well as the comparative experiments on the drivable area and the verification experiments on the navigation line. Section 4 summarizes the results, limitations, and future research directions of the study.

## 2. Materials and Methods

The orchard path navigation recognition method proposed in this paper consists of two parts: drivable area segmentation and navigation line recognition. The drivable area segmentation is based on the U-Net fully convolutional neural network to identify the drivable area in the orchard and obtain the corresponding mask area. The navigation line recognition, based on the semantic segmentation results, uses preprocessing such as HSV conversion, morphological processing, Gaussian blur, and edge smoothing to extract contours and center points, and then performs interpolation optimization processing to recognize the orchard navigation lines.

### 2.1. Image Acquisition and Dataset Creation

**Figure 1:**
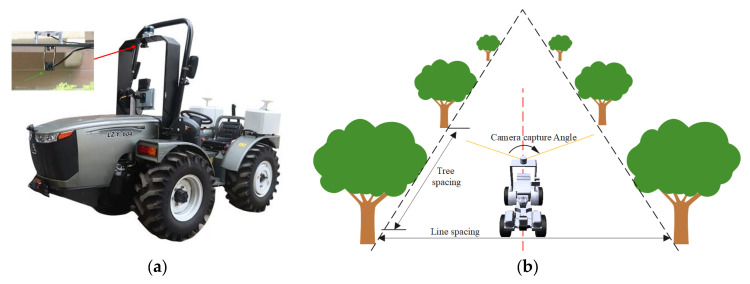
Dataset collection. (**a**) Camera installation position; (**b**) The acquisition angle of the camera.

The data collection site is located in Yantai City, Shandong Province, China. The weather was sunny and the sunlight was abundant, which ensured the quality of the data collection. The data set was collected using the LZY 604 wheeled tractor (Luzhong Tractor Co., Ltd., Weifang, Shandong, China) as the test platform. The AR monocular camera produced by Leopard Imaging Company (Fremont, CA, USA) was installed on the tractor. The installation position of the camera is shown in Figure 1a. The collection angle of the camera is shown in Figure 1b. The tractor moved at a speed of 1.8 m/s in the orchard to obtain the video of the orchard roads.

**Figure 2:**
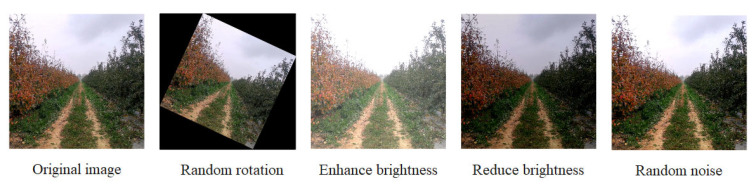
Data augmentation.

The obtained video of the orchard was extracted into 1847 frames through the video frame extraction method to create a dataset. The dataset was divided into a training set and a validation set in a 9:1 ratio. The drivable area was labeled using Labelme [11], and the labels were divided into two categories: the drivable area in the orchard and the background. The drivable area in the orchard was labeled, while the background was not labeled. To expand the data volume of the training samples and improve the accuracy and generalization ability of the model, before training the dataset, this paper used the ImageAug library to perform random data augmentation on the training set images, such as horizontal flipping, adjustment of brightness and saturation, and Gaussian blur, in order to improve the model’s generalization ability to the orchard environment. The data augmentation is shown in Figure 2.

### 2.2. Apparatus for Measuring the Usable Area of Orchards Based on U-Net

**Figure 3:**
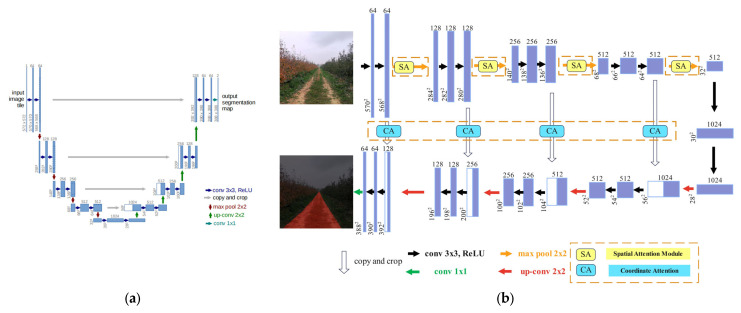
Overall network structure. (**a**) Original U-Net network structure; (**b**) Improved U-Net network structure.

U-Net is an end-to-end image segmentation model based on convolutional neural networks [12], consisting of an encoder for extracting feature information and a decoder for reconstructing the feature images and generating segmented images. The original U-Net network is shown in Figure 3a. The improved U-Net network retains the encoder and decoder structures and skip connections of the original U-Net, and adds spatial attention mechanisms during downsampling, and coordinate attention mechanisms during skip connections. The overall network structure of the improved U-Net is shown in Figure 3b. Traditional models process images uniformly and do not distinguish the importance of regions, ignoring some regions that contain more critical information, resulting in limited performance [13].

#### 2.2.1. Detailed Network Structure Design

The improved U-Net consists of an encoder, decoder, and skip connections. Each stage of the encoder includes two 3 × 3 convolution layers with ReLU activation and a 2 × 2 max pooling operation for downsampling. The number of feature channels is set to 64, 128, 256, 512, and 1024 from shallow to deep layers. This design follows the principle of gradually increasing feature maps to capture higher-level semantic information while reducing spatial resolution, which balances model complexity and feature richness.

In the decoder, each stage performs 2× upsampling followed by two 3 × 3 convolution layers and concatenation with the corresponding encoder features. The number of feature channels decreases symmetrically (512, 256, 128, 64) to progressively recover spatial details.

The Spatial Attention module is inserted after each encoder stage to emphasize critical spatial regions and suppress background noise. The Coordinate Attention module is added to each skip connection to embed position-sensitive information into channel attention, enhancing feature fusion between encoder and decoder without significantly increasing computation.

#### 2.2.2. Spatial Attention Mechanism

**Figure 4:**
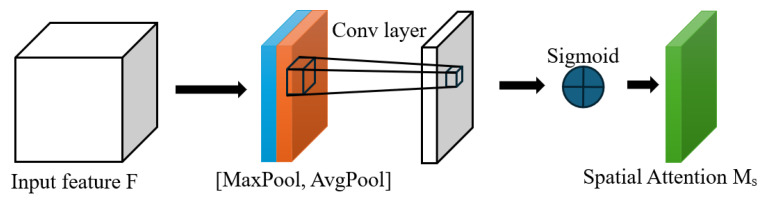
Structure of the spatial attention mechanism.

When traditional models process images, they often uniformly treat the entire image without distinguishing the importance of different regions. This uniform processing approach ignores the fact that certain regions in the image may contain more crucial information, thereby limiting the performance of the model when processing images. The Spatial Attention mechanism emphasizes the regions that contribute the most to the task by weighting specific spatial positions in the feature map [14], while suppressing irrelevant or redundant regions, thereby improving the model’s performance. The structure of the Spatial Attention mechanism is shown in Figure 4. Traditional models may lose local key information during downsampling. The Spatial Attention mechanism, during downsampling, generates a spatial weight map to enable the model to enhance the features of important regions and weaken the influence of irrelevant regions, thus retaining core information while reducing the size.

First, perform global max pooling and global average pooling on the input feature map F with dimensions H × W × C, resulting in two feature maps of size H × W × 1. Then, concatenate the results of global max pooling and global average pooling along the channels to obtain a feature map of size H × W × 2. Finally, perform convolution on the concatenated result to obtain a feature map of size H × W × 1, and apply the Sigmoid activation function to obtain the spatial attention weight matrix Ms. The calculation formula for spatial attention is as shown in Equation (4).

(1) $$M_{s}\left(F\right)\in R^{H,W}$$

(2) $$F_{avg}^{s}\in R^{1\times H\times W}$$

(3) $$F_{max}^{s}\in R^{1\times H\times W}$$

(4) $$\begin{array}{c} M_{s}\left(F\right)=\sigma (f^{7\times 7}([\mathrm{AvgPool}(F);\mathrm{MaxPool}(F)]))\\ =\sigma (f^{7\times 7}[F_{\mathrm{avg}}^{s};F_{\max}^{s}]) \end{array}$$

Among them, $F_{\mathrm{avg}}^{s}$ represents the result of performing global average pooling on the input features, $F_{\max}^{s}$ represents the result of performing global max pooling on the input features, σ represents the Sigmoid activation function, and $f^{7\times 7}$ represents the 7 × 7 convolution operation.

#### 2.2.3. Coordinate Attention Mechanism

**Figure 5:**
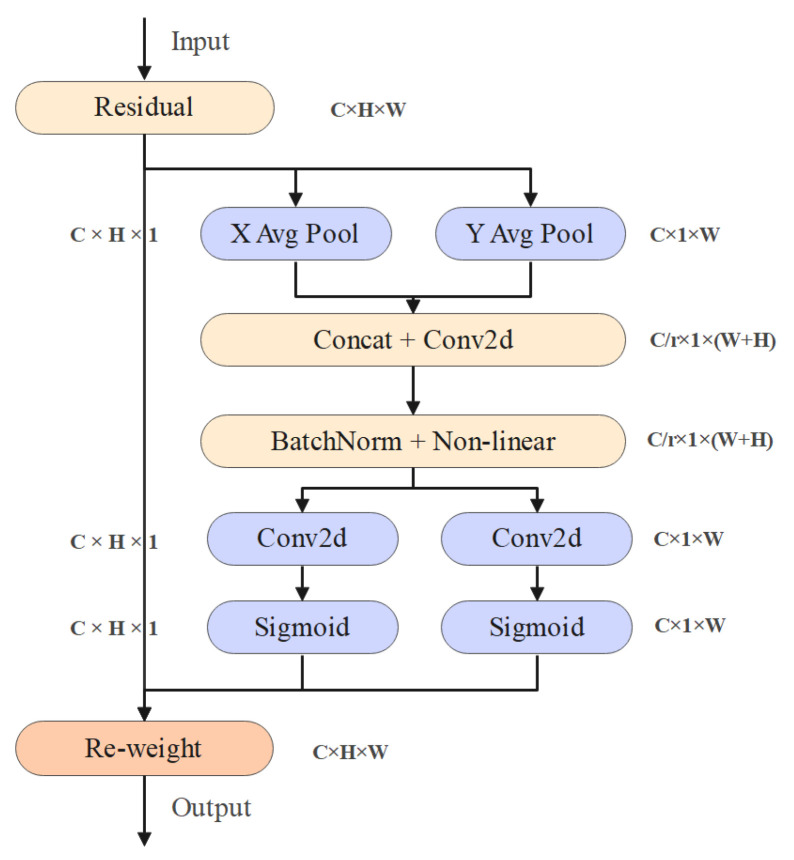
Structure of the Coordinate Attention Mechanism.

The Coordinate Attention Mechanism is achieved by performing average pooling in both the horizontal and vertical directions [15]. It not only captures information across channels but also captures direction-aware and position-sensitive information, which helps the model to more accurately locate and identify the objects of interest. The structure of the Coordinate Attention Mechanism is shown in Figure 5.

CA first divides the input feature map into two directions: height and width, and performs global average pooling separately in each direction to obtain a vertical feature vector $z_{c}^{h}$ of size C × H × 1, as well as a horizontal feature vector $z_{c}^{w}$ of size C × 1 × W, as shown in Equations (5) and (6).

(5) $$z_{c}^{h}(h)=\frac{1}{W}\sum_{0\leq i<W}\;x_{c}(h,i)$$

(6) $$z_{c}^{w}(w)=\frac{1}{H}\sum_{0\leq f<H}\;x_{c}(j,w)$$

Then, the two pooled feature maps are concatenated along the channel dimension to obtain a feature map with dimensions of C × 1 × (W + H). A 1 × 1 convolution is applied to the concatenated feature map for dimensionality reduction, as shown in Equation (7).

(7) $$Z=K_{1}*Y\in R^{\frac{C}{r}\times 1\times (W+H)}$$

Among them, the convolution kernels $K_{1}\in R^{C\times \frac{C}{r}\times 1\times 1}$ and *r* are the scaling factors, and ∗ represents the convolution operation.

Then, normalization processing is carried out, and the formula is as shown in Equation (8).

(8) $$Z_{bn}=\mathrm{BatchNorm}\left(Z\right)=\gamma \cdot \frac{Z-\mu }{\sqrt{\sigma^{2}+\epsilon }}+\beta$$

Here, μ represents the mean difference, $\sigma^{2}$ represents the variance, γ, β represents the learnable parameters, and ϵ represents the small constant to avoid division by zero.

Meanwhile, a nonlinear function h-swish is introduced to enhance the model’s expressive power. The expression of the h-swish function is shown in Equation (9).

(9) $$h-\mathrm{swish}\left(x\right)=x\cdot \frac{\min\left[\max\left(x+3,0\right),6\right]}{6}$$

Finally, the feature maps that have undergone normalization and nonlinear activation are re-segmented into two branches in the vertical and horizontal directions. Then, the outputs of these two branches are respectively applied with the Sigmoid activation function to compress the output values to the range of 0–1. Subsequently, the obtained two attention weight maps are respectively multiplied with the original input feature maps in the corresponding dimensions. This achieves the weighted operation on the original feature map, thereby enhancing the important features and suppressing the non-important ones.

### 2.3. Navigation Line Calculation Method

**Figure 6:**
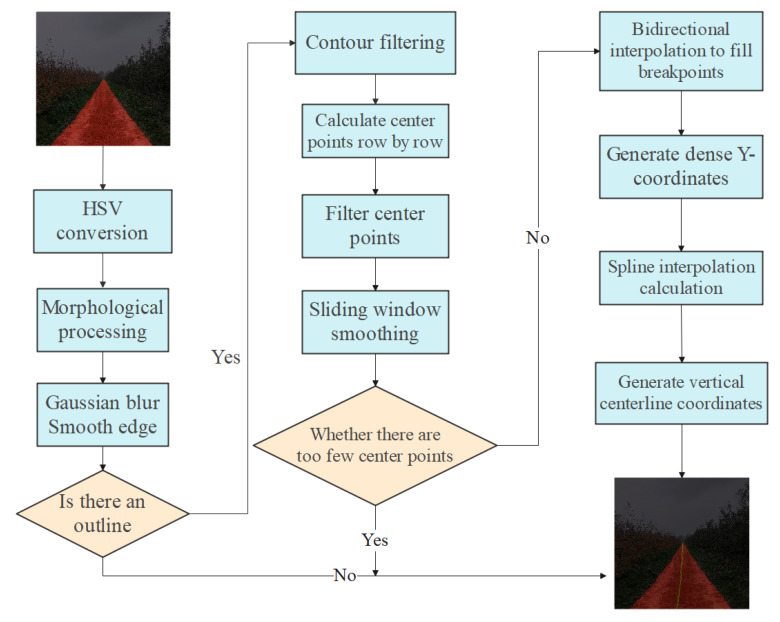
Flowchart of route calculation process.

The existing methods for calculating navigation lines mostly employ Hough transform and vertical projection for linear fitting, which are suitable for structured road sections [16]. However, in orchard environments, the road edges are irregular, and directly calculating navigation lines based on the segmentation results will result in significant errors. In this paper, firstly, the image undergoes HSV conversion, morphological processing, Gaussian blurring, and edge smoothing for preprocessing. Then, the contours and center points are calculated. Finally, interpolation optimization processing is carried out. The flowchart of navigation line calculation is shown in Figure 6.

#### 2.3.1. Image Preprocessing

Image preprocessing lays the foundation for the subsequent calculation of navigation lines, mainly including HSV conversion, morphological processing, and Gaussian blur for edge smoothing operations.

1. HSV Conversion

In the field of computer vision, the RGB color space is intuitive but is greatly affected by changes in lighting, which is not conducive to the subsequent calculation of navigation lines. Through the HSV conversion, the coupling problem between the RGB channels can be avoided. The HSV conversion is to convert the color space of the image from RGB to HSV. The HSV color space is more in line with human perception of colors [17]. When $\max\left(\frac{R}{255},\frac{G}{255},\frac{B}{255}\right)=\min\left(\frac{R}{255},\frac{G}{255},\frac{B}{255}\right)$, the hue H is 0°. When the value of $\max\left(\frac{R}{255},\frac{G}{255},\frac{B}{255}\right)$ is equal to $\frac{R}{255}$, $\frac{G}{255}$, $\frac{B}{255}$, the hue H calculation formulas are as shown in Equations (10)–(12). When $\max\left(\frac{R}{255},\frac{G}{255},\frac{B}{255}\right)\neq 0$, the saturation S calculation is as shown in Equation (13). When $\max\left(\frac{R}{255},\frac{G}{255},\frac{B}{255}\right)=0$, the saturation S = 0. The calculation formula for luminance V is as shown in Equation (14).

(10) $$H=60{}^{\circ}\times \left[\frac{\left(G-B\right)/255}{\max\left(\frac{R}{255},\frac{G}{255},\frac{B}{255}\right)-\min\left(\frac{R}{255},\frac{G}{255},\frac{B}{255}\right)}mod6\right]$$

(11) $$H=60{}^{\circ}\times \left[\frac{\left(B-R\right)/255}{\max\left(\frac{R}{255},\frac{G}{255},\frac{B}{255}\right)-\min\left(\frac{R}{255},\frac{G}{255},\frac{B}{255}\right)}+2\right]$$

(12) $$H=60{}^{\circ}\times \left[\frac{\left(R-G\right)/255}{\max\left(\frac{R}{255},\frac{G}{255},\frac{B}{255}\right)-\min\left(\frac{R}{255},\frac{G}{255},\frac{B}{255}\right)}+4\right]$$

(13) $$S=\frac{\max\left(\frac{R}{255},\frac{G}{255},\frac{B}{255}\right)-\min\left(\frac{R}{255},\frac{G}{255},\frac{B}{255}\right)}{\max\left(\frac{R}{255},\frac{G}{255},\frac{B}{255}\right)}$$

(14) $$V=\max\left(\frac{R}{255},\frac{G}{255},\frac{B}{255}\right)$$

2. Morphological Processing

After the HSV conversion, the image noise is removed through a combined morphological processing of dilation and erosion. The small noise in the image can be eliminated through the erosion operation, and the internal small holes can be filled through the dilation operation. In this paper, the image is processed using the opening operation, which is the sequence of erosion followed by dilation. The formula of the opening operation is shown in Equation (15).

(15) $$f\circ B(x,y)=\underset{(s,t)\in B}{max}\;\underset{(s',t')\in B}{min}\;f\left(x+(s+s'),y+(t+t')\right)$$

Among them, f represents the input image; B represents the structural element; ∘ represents the opening operation; (s,t) and (s',t') represent the pixel coordinate offsets within B.

After the opening operation, smaller noise blocks can be eliminated, while the integrity of the operational area is maintained to facilitate subsequent operations.

3. Gaussian Blur Smooths the Edges

Gaussian blurring is achieved by convolving the image with a Gaussian kernel. The Gaussian kernel function is shown in Equation (16).

(16) $$G(x,y)=\frac{1}{2\pi \sigma^{2}}e^{-\frac{x^{2}+y^{2}}{2\sigma^{2}}}$$

Among them, σ represents the degree of control.

After applying the Gaussian blur operation for edge smoothing, the edge transitions become more natural, avoiding misjudgment in centerline calculation due to sharp edges, laying the foundation for accurately obtaining the navigation line coordinates, and enhancing the stability and reliability of subsequent processing.

#### 2.3.2. Calculation of Navigation Lines

After the image undergoes image preprocessing, the calculation of navigation lines requires precisely extracting the key information of the contour and processing the center point data [18]. In this paper, Canny is used to detect the edge of the drivable area, calculate the boundary contour of the drivable area, and eliminate the background contour and interfering contours. The center points of the filtered target contours are calculated row by row. The formula for calculating the x-coordinate of the center point is as shown in Equation (17).

(17) $$x_{c}(y)=\frac{x_{min}(y)+x_{max}(y)}{2}$$

Among them, $x_{c}(y)$ represents the horizontal coordinate of the center point of the drivable area in the row where the vertical coordinate of the image is y; $x_{min}(y)$ represents the horizontal coordinate of the left boundary of the drivable area within this row; $x_{max}(y)$ represents the horizontal coordinate of the right boundary of the drivable area within this row.

The initial center point sequence may be affected by noise, discontinuous contours, etc., and further filtering and optimization of abnormal points are required. The formulas for calculating the mean μ and standard deviation σ of the center point sequence are respectively shown in Equations (18) and (19).

(18) $$\mu =\frac{1}{m}\sum_{i=1}^{m}\;x_{ci}$$

(19) $$\sigma =\sqrt{\frac{1}{m}\sum_{i=1}^{m}\;(x_{\mathrm{ci}}-\mu {)}^{2}}$$

Here, *m* represents the initial number of center points.

Remove the abnormal center points that meet the $|x_{ci}-\mu |>3\sigma$ criteria, and retain the reasonably distributed center points. Construct a sliding window of size ω=5, and perform sliding average processing on the center point sequence. The formula for the smoothed coordinates of the *i*-th center point within the window is shown in Equation (20).

(20) $${\hat{x}}_{ci}=\frac{1}{\omega }\sum_{j=i-\lfloor \omega /2\rfloor }^{i+\lfloor \omega /2\rfloor }\;x_{cj}$$

Among them, ${\hat{x}}_{ci}$ represents the output value of the *i*-th center point after being smoothed by the sliding window; ω represents the size of the sliding window; $x_{cj}$ represents the original sequence of center points; *i* represents the current center point being processed; *j* represents the center point being traversed within the window.

After being smoothed through the sliding window, the distribution of the center points becomes more uniform, reducing the influence of noise and providing a reliable foundation for subsequent processing.

#### 2.3.3. Generation of Navigation Lines

Based on the optimized sequence of center points, continuous and smooth navigation lines are generated through interpolation fitting. If there are discontinuities in the center point sequence, bidirectional interpolation is used to fill the breakpoints. The interpolation formula is shown in Equation (21). The interpolation results are verified from two directions to ensure the continuity of coordinates at the breakpoints, and a complete sequence of center points is obtained.

(21) $$x(y)=x_{a}+\frac{x_{b}-x_{a}}{y_{b}-y_{a}}(y-y_{a})$$

Here, ($x_{a}-y_{a}$) and ($x_{b}-y_{b}$) represent the adjacent valid center points at the discontinuity points.

To improve the accuracy of the navigation line fitting, based on the original central point’s vertical coordinate, a dense Y coordinate sequence $Y=\{y_{1},y_{1}+\Delta y,y_{1}+2\Delta y,\ldots ,y_{m'}\}$ was generated with a fixed step size, and corresponding horizontal coordinates were supplemented to prepare for spline interpolation. Using cubic spline interpolation, the interpolation function was set as S(y), which satisfied $\left[y_{i},y_{i+1}\right]$ within the region $S(y)=a_{i}+b_{i}(y-y_{i})+c_{i}(y-y_{i}{)}^{2}+d_{i}(y-y_{i}{)}^{3}$, and met the boundary conditions. By solving the spline coefficients $a_{i},b_{i},c_{i},d_{i}$, the horizontal coordinates corresponding to the dense Y coordinates were interpolated to obtain a smooth X coordinate sequence $X=\{S(y_{1}),S(y_{1}+\Delta y),\ldots ,S(y_{m'})\}$.

The dense coordinate pairs obtained from the interpolation (denoted as $\left\{\left(X_{1},Y_{1}\right),\left(X_{2},Y_{2}\right),\ldots ,\left(X_{k},Y_{k}\right)\right\}$, where k represents the number of dense coordinates) are the navigation line coordinates of the exercisable area.

## 3. Experiments and Result Analysis

Improving the U-Net network under the Windows 10 operating system, with a GPU (Quadro P2200) and the code training platform being Pycharm 2020.1. The specific experimental environment is shown in Table 1.

### 3.1. Evaluation Index

This study comprehensively evaluated the network model using multiple quantitative indicators: Recall, Precision, mPA, and mIoU. These indicators measure the performance of the model from different perspectives. Recall reflects the model’s ability to identify positive samples of various categories [19], and the calculation formula is shown in Equation (22). Precision indicates the accuracy of the predicted samples [20], and the calculation formula is shown in Equation (23). Both of these are evaluated from the basic classification layer. mPA and mIoU focus on the characteristics of the segmentation task. mPA is the average classification accuracy of the model across all categories, measuring the consistency and accuracy of classification across various categories, and the calculation formula is shown in Formula (24). mIoU is the average value of the overlap between the predicted regions and the true regions for all categories [21], reflecting the overall segmentation accuracy of the model, and the calculation formula is shown in Formula (25). Inference speed represents the number of inferences the model can complete per second, directly reflecting the model’s efficiency in practical application scenarios, especially in real-time processing tasks. Number of parameters indicates the scale of the model in millions, which is closely related to the model’s complexity, storage requirements, and computational cost.

Through the synergy of these six indicators, both the segmentation performance, the efficiency of the model during inference, and the complexity reflected by the number of parameters are considered, ensuring the feasibility and effectiveness of the model in practice.

(22) $$Recall=\frac{P_{ii}}{P_{ii}+P_{ij}}$$

(23) $$Precision=\frac{P_{ii}}{P_{ii}+P_{ji}}$$

(24) $$mPA=\frac{1}{k+1}\sum_{i=0}^{k}\;\frac{P_{ii}}{\sum_{j=0}^{k}\;P_{ij}}\times 100\%$$

(25) $$mIoU=\frac{1}{k+1}\sum_{i=0}^{k}\;\frac{P_{ii}}{\sum_{j=0}^{k}\;\left(P_{ij}+P_{ji}\right)-P_{ii}}\times 100\%$$

Here, *k* represents the total number of categories including the background in the class; *i* represents the drivable area category, and *j* represents the non-drivable area category; $P_{ii}$ is the number of pixels that are actually and predictively classified as category *i*; $P_{ij}$ is the number of pixels that are actually classified as category *i* but predicted as category *j*; $P_{ji}$ is the number of pixels that are actually classified as category *j* but predicted as category *i*.

### 3.2. Model Training

**Figure 7:**
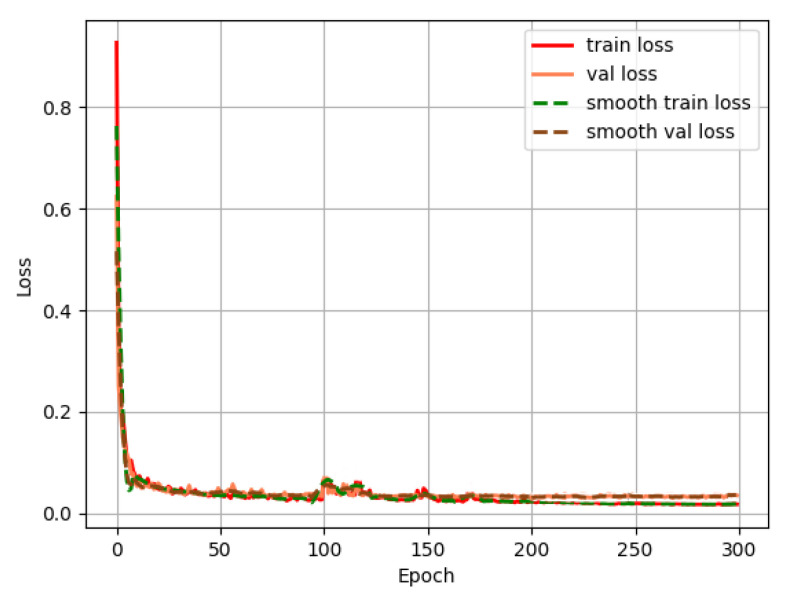
Curve showing the variation of loss value with the number of training rounds.

During the model training process, the loss value is a key indicator for measuring the learning effect of the model. By monitoring the changes of training loss and validation loss with respect to the training epochs, one can intuitively understand the development trend of the model’s fitting and generalization capabilities. The curve of the loss value changing with the training epochs is shown in Figure 7.

Both the train loss and val loss decreased rapidly, indicating that in the initial training rounds, the model quickly learned the data patterns, reduced the prediction error, and significantly improved its fitting ability for the task. During the middle stage (Epoch 50–200): The loss decreased at a slower rate, and the fluctuations in the train loss became smaller. The val loss occasionally had small fluctuations (around 100 epochs), reflecting that the model was gradually approaching the optimal solution. At the same time, the data distribution in the validation set differed from that in the training set, causing fluctuations in the validation loss. In the later stage (Epoch 200–300): Both the train loss and val loss tended to stabilize. The values were close and the fluctuations were extremely small, indicating that the model had fully learned the data features and entered the “convergence plateau period”. From the smooth train loss and smooth val loss, it can be more clearly observed: The two smooth curves have a highly consistent trend, and eventually approached around 0.04, indicating that the model training process was stable and the convergence effect was good. The curve was flat in the later stage, proving that the model had learned stable feature representations.

### 3.3. Model Performance Verification

To verify the completeness of the information on drivable areas and the accuracy of the navigation lines obtained by the method proposed in this paper in an orchard environment, comparative experiments on drivable area detection and verification experiments on navigation line calculation were conducted respectively.

#### 3.3.1. Comparison Validation

To verify the effectiveness of the model proposed in this paper, under the same configuration environment, the U-Net, SegViT, SE-Net and DeepLabv3+ deep learning network models were selected to conduct comparative experiments on the dataset of this paper. The performance indicators of each model are compared as shown in Table 2.

**Figure 8:**
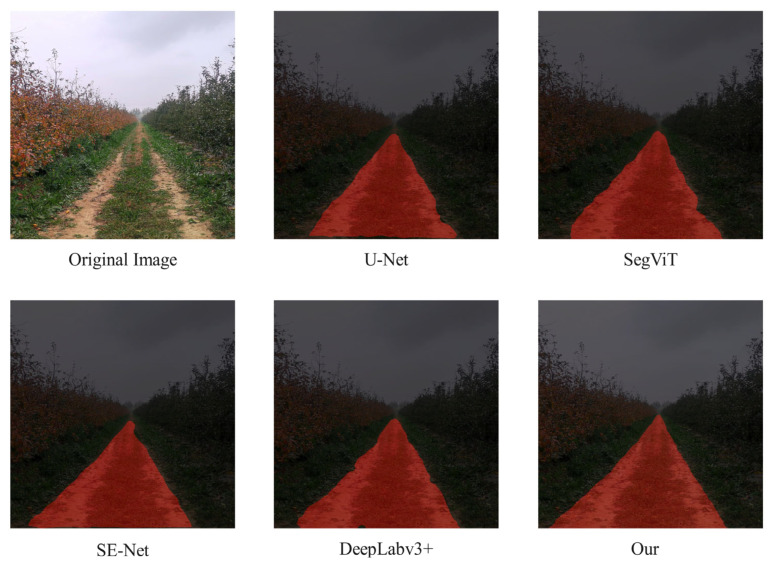
Comparison of visualization results.

Figure 8 shows the segmentation visualization results of each model on the test set. From Figure 8, it can be seen that most models have incomplete recognition of the drivable areas. The improved U-Net model has the best recognition effect, and can obtain more complete and accurate drivable areas in the orchard, with higher segmentation accuracy.

**Figure 9:**
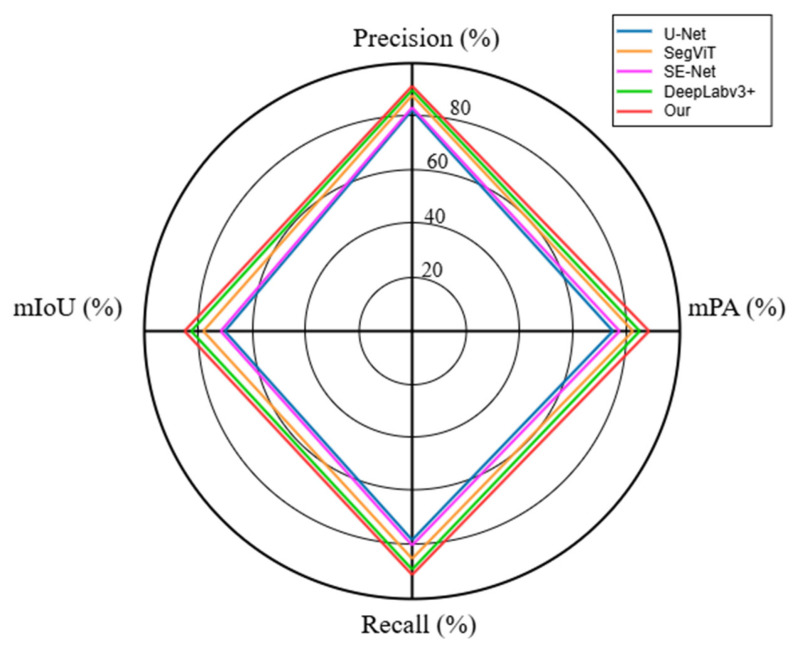
Model comparison radar chart.

To present the comparison results more clearly, the model comparison radar chart is shown in Figure 9. The radar chart presents the values of the four different indicators corresponding to the models summarized in Table 2. Each axis of the radar chart corresponds to one of the indicators. The shapes formed by the lines show the performance capabilities and shortcomings of each model in the multi-dimensional space. The value range of all indicators is between 0% and 100%. The closer the value is to 100%, the better the result. From Figure 9, it can be seen that the results of all models fall within a similar range. The improved U-Net method proposed in this paper demonstrates outstanding performance.

#### 3.3.2. Navigation Line Verification

**Figure 10:**
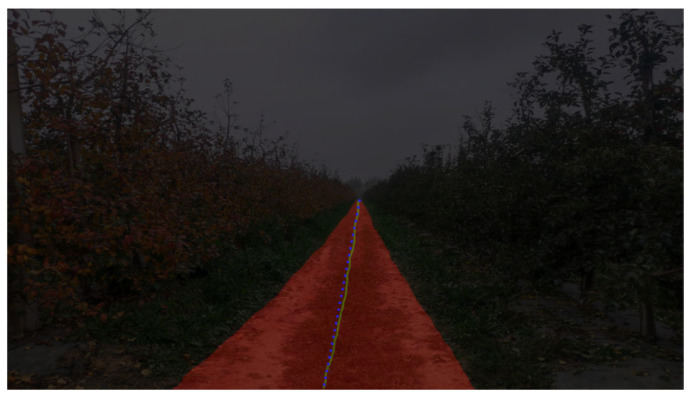
Comparison of calculated navigation line and manually observed navigation line. Note: The (yellow) line in the figure is the fitting navigation line, and the (blue) line is the manual observation navigation line.

To meet the requirements of agricultural machinery and agricultural techniques. For large-scale and standardized orchards, the error of the navigation line needs to be controlled within ±50 to 80 mm. Otherwise, it may affect the connection of subsequent mechanized harvesting and other links. The error within a reasonable range can meet the safety operation requirements of the orchard. The comparison of the navigation line calculated and the navigation line observed manually is shown in Figure 10. The manually observed navigation line is used as the standard to judge the accuracy of the extraction of the orchard navigation line. The mean absolute error of the calculated navigation line compared with the manually observed navigation line is 56 mm, and the standard deviation of the error is 4.47 mm.

**Figure 11:**
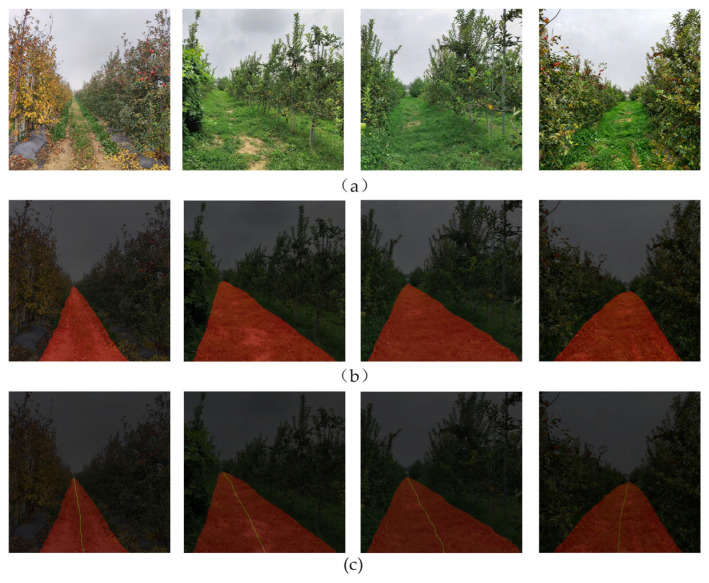
Experimental results. (**a**) Original image; (**b**) Segmentation result of the drivable area; (**c**) Navigation line calculation result.

To further illustrate the effectiveness of the proposed method, Figure 11 shows the visualization of the original image, the drivable area detection, and the corresponding navigation line calculation results.

## 4. Discussion

This study addresses the core issue of complex image backgrounds and numerous interfering factors in visual navigation of orchards. It proposes a method for segmented driving area and geometric center-based navigation path recognition based on the improved U-Net. The effectiveness and practicality of the method have been verified through experiments. The CA and SA modules are introduced on the basis of the traditional U-Net. The SA module is inserted in the downsampling stage to enhance spatial feature extraction, and the CA module is added in the skip connection stage to supplement context information. This achieves precise segmentation of complex orchard environments containing bare soil, obstructions, trees, and weeds. The experimental results show that the Recall of the improved model is 90.23%, the Precision is 91.71%, the mPA is 87.75%, and the mIoU is 84.84%. In the same environment, all indicators are superior to those of traditional U-Net, SegViT, SE-Net, and DeepLabv3+. This effectively reduces misjudgments caused by environmental interference and lays a high-quality segmentation foundation for navigation path recognition. Based on the segmented driving area results, the mask image is optimized through HSV conversion, morphological opening operation, Gaussian blur, etc. Combined with contour detection, geometric center calculation, 3σ outlier elimination, sliding window smoothing, and bidirectional interpolation filling, the generated continuous smooth navigation lines have an average distance error of only 56 mm from the actual center line of manual observation, fully meeting the agronomic requirements of ±50–80 mm for mechanized operations in large-scale and standardized orchards. It can ensure the safety and process connectivity of mechanized operations such as picking, spraying, and weeding in the orchard. Under the current orchard environment and hardware configuration, the method proposed in this study can provide effective technical references for the visual navigation task of agricultural machinery in orchards. However, there are still deficiencies, and further optimization is needed in subsequent work. There are limitations in environmental adaptability. The performance of the method in extreme environments has not been fully verified. The dynamic response of path planning is insufficient. This study did not consider dynamic interference factors in orchard operations. Based on the development trend of smart agriculture and the shortcomings of this study, subsequent research will be conducted in the following directions to further improve the orchard visual navigation technology system. Enhance multi-scenario adaptive ability. Expand experimental scenarios and datasets to ensure the stability of the method in different scenarios. Integrate dynamic path planning and multi-sensor data. Introduce sensors such as laser radar, millimeter-wave radar, etc., and combine visual data for multimodal fusion to achieve real-time adjustment of navigation lines and solve the problem of path avoidance under dynamic interference in orchards, improving the flexibility of autonomous operation of agricultural machinery.

## 5. Conclusions

This study proposes an improved method based on U-Net for navigable area segmentation and geometric center-based navigation path recognition, which is applied to orchard visual navigation. The improved U-Net network emphasizes key regions and suppresses redundancy during downsampling by weighting specific spatial positions in the feature maps. This approach improves the network’s performance. In contrast, the traditional U-Net simply concatenates features during feature extraction. The improved U-Net, however, introduces the CA module without increasing model parameters or computational cost. This module effectively fuses the global information obtained during feature extraction, optimizing the feature restoration process of the drivable area in the orchard. As a result, it reduces environmental interference and further improves the overall segmentation accuracy of the model. Although this method provides an effective technical reference for the visual navigation of agricultural machinery in current conditions, it has limitations in environmental adaptability and dynamic path planning response. Future research will focus on enhancing multi-scenario adaptability by expanding experimental scenarios and datasets, and integrating dynamic path planning with multi-sensor data fusion to improve the flexibility of autonomous operation of agricultural machinery.

## Figures and Tables

**Table 1:** Specific Experimental Environment.

Item	Content
Operating System	Windows 10
CPU	Intel(R) Core(TM)i7-10700F CPU @ 2.90 GHZ (Intel®: Santa Clara, CA, USA)
GPU	NVIDIA Quadro P2200 (NVIDIA: Santa Clara, CA, USA)
Platform Environment	Pycharm 2020.1, Pytorch 1.10.0, opencv 4.10.0, Cuda 10.1 with cudann

**Table 2:** Comparison of performance indicators for each model.

Method	Recall/(%)	Precision/(%)	mPA/(%)	mIoU/(%)	Inference Speed/(FPS)	Number of Parameters/(M)
U-Net	78.17	82.57	75.16	70.40	38.5	31.1
SegViT	85.75	88.74	82.90	78.15	46.7	38.6
SE-Net	80.06	84.19	77.12	73.36	37.8	36.7
DeepLabv3+	88.24	90.05	85.64	82.89	53.3	62.4
Our	90.23	91.71	87.75	84.84	72.2	45.7

## Data Availability

The data presented in this study are available on request from the corresponding author.
